# *Salmonella enterica* Subsp. *houtenae* Associated with an Abscess in Young Roe Deer (*Capreolus capreolus*)

**DOI:** 10.3390/pathogens10060654

**Published:** 2021-05-25

**Authors:** Adriana Trotta, Laura Del Sambro, Michela Galgano, Stefano Ciccarelli, Erika Ottone, Domenico Simone, Antonio Parisi, Domenico Buonavoglia, Marialaura Corrente

**Affiliations:** 1 Department of Veterinary Medicine, University of Bari “Aldo Moro”, Str. Prov. per Casamassima Km 3, 70010 Valenzano, BA, Italy; adriana.trotta@uniba.it (A.T.); michela.galgano@uniba.it (M.G.); stefano.ciccarelli@uniba.it (S.C.); domenico.buonavoglia@uniba.it (D.B.); 2 Istituto Zooprofilattico Sperimentale della Puglia e Basilicata, Sezione di Putignano, Contrada San Pietro Piturno, 70017 Putignano, BA, Italy; laura.delsambro@hotmail.it (L.D.S.); dome.simone@gmail.com (D.S.); parisi.izspb@gmail.com (A.P.); 3 Parco Nazionale Pollino, Complesso Monumentale Santa Maria della Consolazione, 85048 Rotonda, PZ, Italy; erikaottone.vet@gmail.com

**Keywords:** extraintestinal salmonellosis, localized lesion, abscess, soft tissue, roe deer, AMR resistance, WGS analyses

## Abstract

Background: *S. enterica* subsp. *houtenae* has been rarely documented, and very limited genomic information is available. This report describes a rare case of primary extraintestinal salmonellosis in a young roe deer, associated with *Salmonella enterica* subsp. *houtenae.* Methods: A traditional cultural-based analysis was carried out from the contents of a neck abscess; biochemical identification and PCR assay were performed to isolate and identify the pathogen. Through whole-genome sequencing (WGS), multilocus sequence typing (MLST), core genome MLST (cgMLST), and the Salmonella pathogenicity islands (SPIs) survey, resistome and virulome genes were investigated to gain insight into the virulence and antimicrobial resistance of *S. houtenae*. Results: Biochemical identification and PCR confirmed the presence of *Salmonella* spp. in the swelling. The WGS analysis identified *Salmonella enterica* subspecies *houtenae* serovar 43:z4,z23:- and ST 958. The virulence study predicted a multidrug resistance pattern with resistance shown against aminoglycosides, tetracycline, beta-lactamase, fluoroquinolones, fosfomycin, nitroimidazole, aminocoumarin, and peptide. Fifty-three antibiotic-resistant genes were identified. No plasmids were detected. Conclusion: This study demonstrates the importance of continuous surveillance of pathogenic salmonellae. Biomolecular analyses combined with epidemiological data can provide important information about poorly described Salmonella strains and can help to improve animal welfare.

## 1. Introduction

Salmonellae are important zoonotic enteropathogens that affect humans, livestock, companion and zoo animals, and wildlife [1]. *Salmonella* spp. is the organism second-most responsible for gastrointestinal human infections after *Campylobacter* spp. In 2018, 91,857 confirmed cases of salmonellosis in humans and 119 deaths in the EU were reported [2]. Based on the 16S rRNA sequence and biochemical analysis, the Salmonella genus includes two species: *S. bongori* and *S. enterica*; the latter is further divided into six different subspecies (I-VI): *enterica*, *salamae*, *arizonae*, *diarizonae*, *houtenae* and *indica*. In contrast with *S. enterica* subsp. *enterica*, for which there are many studies about prevalence, antimicrobial resistance, virulence, infection ability, and biofilm formation [3], very limited genomic information is available about *S. houtenae. Salmonella enterica* subsp. *houtenae* was originally isolated in 1978, and currently comprises 73 distinct serovars [4], which have been recovered from a variety of animals, including mammals, birds, reptiles, and amphibians [4]. In addition, *S. enterica* subsp. *houtenae* has rarely been documented in human samples (<1% of isolates) in association with meningitis, brain abscesses, or extraintestinal syndrome, primarily affecting children and immunocompromised adults [4]. The extraintestinal syndrome is characterized by the localization of a Salmonella infection mostly in soft tissue (skin or muscles). The skin is the most common infection site, and lesions vary from pustular dermatitis to severe deep cellulitis and abscesses. A variety of wild species, such as hedgehogs [5], wild birds [6], reptiles [7,8], wild mammals [6], and white-tailed deer [9], show a key role in the epidemiology of salmonellosis as asymptomatic carriers and intermittent shedders of a broad range of Salmonella serotypes [9]. The prevalence of Salmonella serotypes in wild animals may vary from species to species depending on many factors, such as age and type of breeding [9]. Elk (*Cervus elaphus nelsoni*), sika deer (*Cervus nippon*), white-tailed deer (*Odocoileus virginianus*), and roe deer (*Capreolus capreolus*) are recognized as potential carrier animals of Salmonella serotypes [9,10]. Nevertheless, young individuals remain the most exposed to clinical disease. It is well-described that gastrointestinal symptoms (e.g., enteritis and diarrhea), especially those associated with specific serovars of *S. enterica*, such as *Salmonella typhimurium*, are a crucial problem during the first few weeks of post-natal life [11]. In these types of subjects, salmonellae may cause septicemia and death. Compared with human data [10], the incidence of the extraintestinal salmonellosis both in wild and domestic species is less-documented, with few reports in horses or foals [12].

In this study, we describe a rare case of primary extraintestinal salmonellosis involving the neck tissues of a young free-ranging roe deer (*Capreolus capreolus*), associated with *Salmonella enterica* subsp. *houtenae*, as a post-surgery complication after bone fracture reduction.

## 2. Case Report

In June 2020, a 7-day-old female roe deer (*Capreolus capreolus*) was referred by the veterinary practitioner at the National Park of Pollino (NPP), located in Southern Italy (Calabria), to the Section of Surgery of the Department of Veterinary Medicine of Bari (DVM) for evaluation of a severe lameness. The roe deer was referred to the NPP as he was probably abandoned by his mother due to his inability to walk independently. Therefore, the young deer had been hand-raised by the practitioner at the NPP with artificial milk.

Clinical evaluation revealed suboptimal body conditions, with mild dehydration status. The great organic functions (GFOs) were found to be normal, and no vomiting or diarrhea were present. Upon orthopedic evaluation, the roe deer had a grade IV lameness and signs of intense pain. Moderate pitting edema was appreciable on palpation of the right-leg swelling, and the swelling was warm to the touch. Radiographic findings showed a multiple fracture.

Hematological tests were performed by the veterinarian at the NPP prior to referral of the subject to the DVM. A blood count revealed a mild leukocytosis, with WBC 11 × 10^3^ cells/L and neutrophilia (6.4 × 10^3^ cells/L). The HCT was 44%, and platelet counts (480 × 10^3^ platelets/L) were within reference limits [13]. Other biochemical parameters were found to be in the normal range.

The young roe deer underwent the surgical stabilization of the multiple fracture after 12 h of fluid therapy with NaCl 0.9% and antibiotic therapy with cephalexin (25 mg/kg IV TID). The subject was stabled in an individual box stall overnight and during the day with manual breastfeeding every 3 h. After the surgery, the roe deer was kept under observation for 48 h in the DVM before being released to the NPP with a rehabilitation protocol. In the follow-up examination, one month after surgery, a neoformation of about 5 cm in diameter appeared on the right side of the neck, adherent to the surrounding tissues, and was hard, painful, and warm. The neoformation was suspected to be an abscess; therefore, the swelling was subjected to an aspiration needle and part of the contents was subjected to microbiological analysis. Surgical curettage, draining the abscess, and inserting passive drainage (Penrose type) were performed during the removal of the bone pins. In addition, a sterile swab immersed in transport media (Nuova APTACA, Brescia, Italy) was collected from the roe deer rectal tract for microbiological examination. The samples were immediately transported to the bacteriology laboratory of the DVM for microbiological investigation.

## 3. Results

### 3.1. Microbiological Investigation

Analysis of the contents of the swelling revealed a massive growth of Gram-negative, lactose-negative bacteria on Columbia blood agar (CBA) (Liofilchem, Teramo, Italy) and MacConkey agar (MCK) (Liofilchem, Teramo, Italy) after 24 h of incubation at 37 °C in aerobic conditions. From the rectal swab, Gram-negative, lactose-positive colonies were seen and identified as *Escherichia coli* with biochemical tests. No growth on MSA plates was recorded from all the examined samples, even after prolonged incubation. Suspected colonies of Gram-negative, lactose-negative bacteria grown on MCK from the swelling and subcultures both on selective and differential media, such as xylose-lysine-desoxycholate (XLD) (Oxoid, Milan, Italy), modified semi-solid rappaport-vassiliadis (MRSV) (Oxoid, Milan, Italy) and triple sugar iron (TSI) (Oxoid, Milan, Italy), showed the typical aspects of *Salmonella* spp., i.e., xylose positivity and H_2_S positivity on XLD, a halo of migration starting from the inoculation point on MRSV and lactose negativity, and H_2_S positivity on TSI. Five different suspected *Salmonella* spp. colonies grown on XLD and TSI and subjected to biochemical identification were found positive for *Salmonella* spp. with an identity score of 99%. In addition, the same pure colonies, tested with PCR assay targeting the *invA* gene, were found positive for *Salmonella* spp. Antimicrobial susceptibility testing revealed that the isolate was resistant to chloramphenicol (CHL; 30 µg) and oxytetracycline (OT; 30 µg), and susceptible to ampicillin (AMP; 10 µg), amoxicillin–clavulanic acid (AMC; 20 µg of amoxicillin + 10 µg of clavulanic acid), gentamicin (GN; 10 µg), enrofloxacin (ENR; 5 µg), cephazolin (CFX; 30 µg), and trimethoprim sulfamethoxazole (SXT; 1.25 µg of trimethoprim + 23.75 µg of sulfamethoxazole).

### 3.2. Genomic Analysis

A single isolated colony from XLD agar was chosen for deeper investigation through whole-genome sequencing (WGS). The genome assembly consisted of 69 DNA contigs amounting to ~4.712 Mbp in size, with a G + C content of 51.69%. The average genome coverage was 93X. Genome analysis through the Salmonella in silico typing resource (SISTR) (available online at https://lfz.corefacility.ca/sistr-app/, accessed on 20 March 2021), identified the isolate as *Salmonella enterica* subspecies *houtenae* serovar 43:z4,z23:- and ST 958, whereas ribosomal multilocus sequence typing (rMLST) putatively assigned the isolate to the taxon *Salmonella enterica*. Genome annotation revealed that the most abundant clusters of orthologous groups (COGs) categories were proteins related to the transport and metabolism of amino acids (270 genes) and energy production and conversion (258 genes). The genes assigned to COG functional categories are shown in Table 1.

The reconstructed genome was further analyzed against the databases ARG-ANNOT (doi:10.1128/AAC.01310-13), NCBI (The NCBI Pathogen Detection Project. Bethesda (MD): National Library of Medicine (US), National Center for Biotechnology Information. 2016 May, available online: https://www.ncbi.nlm.nih.gov/pathogens/, accessed on 25 March 2021), AMRFinderPlus (available online: https://doi.org/10.1128/AAC.00483-19, accessed on 25 March 2021), CARD (available online: doi:10.1093/nar/gkw1004, accessed on 25 March 2021), and ResFinder (available online: doi:10.1093/jac/dks261, accessed on 25 March 2021) using the ABRicate tool (Seemann T, Abricate, Github, available online: https://github.com/tseemann/abricate, accessed on 25 March 2021). In total, 53 antibiotic-resistant gene determinants from eight different antibiotic classes were identified (Table 2).

The databases predicted antimicrobial resistance (AMR) to aminoglycosides, tetracycline, beta-lactamase, fluoroquinolones, fosfomycin, nitroimidazole, aminocoumarin, and peptide. Moreover, 27 genes for multidrug resistance were identified. One hundred and seventy-eight virulence genes were predicted using the Virulence Factors Database [14], including those from several Salmonella pathogenicity islands (Table 3). No plasmids were detected using the Plasmid Finder database [15].

## 4. Discussion

In humans, soft-tissue Salmonella infections are thought to be secondary to bacteremia resulting from colonization of the gastrointestinal tract by Salmonella organisms, but only a limited number of affected individuals have a history of vomiting or diarrhea, or of having *Salmonella* spp. isolated from their feces [16]. Moreover, muscle infections (pyomyositis, abscess, myonecrosis, or necrotizing fasciitis) caused by *Salmonella* spp. seem to be uncommon and may be more frequent in individuals with specific risk factors (i.e., immunosuppression, HIV infection, diabetes, sickle-cell anemia, and aplastic anemia).

*S. houtenae* (IV) is a rare subspecies, comprising less than 1% of all Salmonella strains [17]; nevertheless, this serovar has been isolated from a variety of animals, including mammals, birds, reptiles, and amphibians, but very limited information is available regarding its pathogenicity [4]. When infecting humans, *S. houtenae* can cause intestinal infection; moreover, other studies showed that this serovar is capable of causing serious infections, including septicemia and abscesses [18]. Interestingly, according to the review by Lamas et al. [4], this subspecies shows a strong affinity for brain tissues, causing meningitis, subdural empyema, and extraintestinal infections.

With the aim of detecting a potential pathogen, we investigated the genetic characteristics of Salmonella AMR and virulence genes, isolated from a young roe deer using both microbiological culture and molecular methods.

Regarding antimicrobial susceptibility, few data are available concerning the AMR of *S.* subspecies *houtenae*; some of these studies reported an increase of AMR in non-typhoidal Salmonella strains over the years. Franco et al. showed a wild-type susceptible phenotype of *S. houtenae* against major classes of antimicrobials used in human therapy [19]. In 2013, it was observed that *S. houtenae* strains had acquired resistance to ampicillin and tetracycline [20].

Traditional first-line treatments for Salmonella infections include antimicrobial agents such as chloramphenicol, ampicillin, and trimethoprim-sulfamethoxazole [21]. In this study, we performed either in silico and in vitro analyses for AMR. The phenotypic test revealed that the isolate was resistant to chloramphenicol and oxytetracycline, and susceptible to ampicillin, amoxicillin-clavulanic acid, gentamicin, enrofloxacin, cephazolin, and trimethoprim-sulfamethoxazole; in accordance, in the assembled draft genome, we detected specific genetic determinants that could induce this resistance except for trimethoprim-sulfamethoxazole. The sequenced isolate showed a genotype susceptible to different antimicrobial families such as aminoglycosides, tetracycline, beta lactamase, and fluoroquinolones. However, resistance was found against other specific determinants such as fosfomycin, nitroimidazole, aminocoumarin, and peptide. Although previous studies reported that phenotypic and genotypic resistances in *Salmonella* spp. are usually highly correlated, some discrepancies were noted between phenotypic and genotypic tests, such as in aminoglycosides and beta-lactams resistance [22]. It is therefore essential to combine genotypic and phenotypic information from our AMR results by continuously updating the databases used for this purpose.

Numerous virulence genes that may be found in various elements of the genome, including integrated bacteriophage DNA, Salmonella pathogenicity islands (SPIs), and Salmonella genomic islands (SGIs), are essential for Salmonella pathogenesis [23]. Salmonella pathogenicity islands (SPIs) are gene clusters located at the large chromosomal DNA region and encode for various virulence factors (adhesion, invasion, toxin genes, etc.) [4]. To date, several SPIs have been reported for different Salmonella serovars, with greater focus on SPI1-5 [23]. Some of the effector proteins of the SPI-1 and SPI-2 secretion system T3SS and type VI (T6SS) within Salmonella pathogenicity island 6 were identified in the isolate; T6SSs are versatile systems that deliver toxins into either eukaryotic or prokaryotic cells [24]. Two different TTSS belonging to SPI5 were also identified. SPI1 was identified as a DNA region, encoding a type III secretion system that transports bacterial proteins, such as the actin-binding protein *SopE*, into the cytosol of the target cell, leading to an uptake of the bacterium by the cell [25]. The SPI2 locus encodes a second type III secretion system, and is required for bacteria survival in both epithelial cells and macrophages [26]. SPI-3 includes 10 open reading frames, amongst which the *mgtC* gene is required for growth in an Mg^2+^-limited environment [27]. SPI-5 is associated with enteropathogenesis [28]. It encodes effectors induced by distinct regulatory cues and targeted to different TTSS; *sopB* is secreted by TTSS of SPI1, and *pipB* is translocated by SPI-2 [28].

Salmonella pathogenicity island 2 (SPI-2) and other mobile elements such as Salmonella virulence plasmid (spv) and *sodCI* genes allow salmonellae to survive inside macrophages and facilitate pathogen spreading through the host body [29]. In the isolate, *spvB* and *sodCI* genes were also found. The *SpvB* gene is present in the majority of strains associated with extra-intestinal infections in humans and animals, while *sodCI* is a phage-associated gene that protects Salmonella against oxidative bursts, and it is generally found in most virulent strains [30].

Among the magnesium transporters, the *mgtCB* locus was found, encoding two proteins: *mgtB*, a 102 kDa P-type ATPase Mg^2+^ transport protein, and *mgtC*, a hydrophobic protein with a molecular mass of 22.5 kDa. The *mgtCB* locus was identified as part of the Salmonella pathogenicity island SPI-3, and permits growth in environments with low concentrations of Mg^2+^ in macrophages [31].

The virulence factors such as *sopB*, *sopD* and *pipD*, identified in the *S. houtenae* isolate, are essential for Salmonella enteropathogenicity, and can cause an acute inflammatory cells influx, secretion of intestinal fluid, and enteritis that correlates with clinical diarrhea [32]. Additionally, the isolate carried *invA*, *iroB*, and *spiC* genes encodes for effectors that induce successful host infection. In particular, the *invA* gene codifies for one of the most studied virulence factors with particular interest in the intestinal virulence [33]; it contains sequences that are unique to the genus Salmonella and thus it is also used as a biomarker for the detection of Salmonella spp. Moreover, *invA* gene detection was carried out using the polymerase chain reaction (PCR) technique and whole-genome sequencing (WGS) analyses.

The *iroB* gene is a member of the *iroA* gene cluster (iroBCDEN), which is essential for iron absorption within the host and is responsible for the synthesis and transport of enterobactin, a siderophore produced by *Salmonella* spp. [34]. In particular, *IroB* shows homology with bacterial glycosyl transferases; its specific role is to encode the glucosyltransferase that glucosylates enterobactin [35].

Another interesting virulence gene harbored by the isolate is represented by *spiC*, located in the SPI-2, which is essential for intracellular survival and host defense escape. The *spiC* gene confers the ability to escape from macrophage defense, mediating the activation of signal transduction pathways [36].

Thin aggregative fimbriae with the related genes *csgA*, *csgB*, *csgC*, *csgE*, *csgF* and *csgG* were also found, which aid in attaching to the villi of enterocytes, which also allows bacteria to aggregate with each other. In general, fimbriae play an important role in the pathogenesis of Salmonella; the *fim* gene cluster (including five genes of our isolate) mediates Salmonella adhesion to host cells, and has been implicated in many other roles such as biofilm formation, cell invasion, seroconversion, hemagglutination, and interactions with macrophages [37].

Lastly, the presence of determinants related to flagella was investigated, and *CheR* and *CheB*, genes were identified. Those genes encode proteins required for chemotaxis [38].

## 5. Material and Methods

### 5.1. Microbiological Investigation

The contents of the swelling (approximately 3 mL) and the rectal swab were cultured on plates of Columbia blood agar (CBA), MacConkey agar (MCK), and Mannitol salt agar (MSA) (Liofilchem, Teramo, Italy) at 37 °C for 48 h and incubated in anaerobic, aerobic, and microaerophilic conditions. Initial presumptive bacterial identification of pure colonies grown on plates was performed by Gram staining and oxidase tests (Liofilchem, Teramo, Italy), and subcultured on selective media for *Salmonella* spp.: xylose lysine desoxycholate agar (XLD, Oxoid, Milan, Italy) and modified semisolid rappaport vassiliadis (MRSV) agar (Oxoid, Milan, Italy) incubated at 37 °C for 48 h. Colonies with typical aspects of Salmonella on XLD were subcultured on differential medium triple sugar iron (TSI) (Oxoid, Milan, Italy) and incubated at 37 °C for 24 h. Oxidase-negative bacteria grown on TSI and XLD, suspected to be *Salmonella* spp., were selected for biochemical identification with an API system (Biomérieux, Marcy l’Étoile, France) and a PCR assay targeting the *invA* gene [39]. In addition, antimicrobial susceptibility testing was performed by the agar diffusion disk method, testing the following antimicrobials (disk abbreviation and contents in brackets): chloramphenicol (CHL; 30 µg), oxytetracycline (OT; 30 µg), ampicillin (AMP; 10 µg), amoxicillin–clavulanic acid (AMC; 20 µg of amoxicillin + 10 µg of clavulanic acid), gentamicin (GN; 10 µg), enrofloxacin (ENR; 5 µg), cephazolin (CFX; 30 µg), and trimethoprim sulfamethoxazole (SXT; 1.25 µg of trimethoprim + 23.75 µg of sulfamethoxazole). The discs were all obtained from a single source (Liofilchem, Teramo, Italy). Clinical and Laboratory Standards Institute [40] breakpoints were used for the interpretation of the tested antimicrobials.

### 5.2. Genomic Analysis

Genomic DNA isolated from a single colony of *Salmonella* spp. was extracted using a DNeasy Blood & Tissue Kit (Qiagen, Hilden, Germany), according to the manufacturer’s protocol. The DNA concentration was estimated by a Qubit Fluorometer using Qubit dsDNA HS Assay (Thermo Fisher Scientific, Waltam MA, USA). A paired-end genomic library was prepared using a Nextera DNA Flex Library Preparation Kit (Illumina, San Diego, CA, USA). Sequencing was performed using a MiSeq Reagent Kit v2 (2 × 250 bp) on an Illumina MiSeq platform (Illumina, San Diego, CA, USA) as described by Bianco et al. [41]. More information regarding the Illumina technology can be found on their website (available online: http://www.illumina.com/?dnr=1, accessed on 5 April 2021). The paired-end raw reads were trimmed using Trimmomatic (v0.36.6) [42] and the novo genome assembly was performed by SPAdes (v3.12.0) [43]. Both tools were run through a Galaxy instance.

In order to provide serovar prediction using a genoserotyping approach, and to define additional sequence-based typing analyses for multilocus sequence typing (MLST) [44] and core genome MLST (cgMLST), the assembly was submitted to the Salmonella in silico typing resource (SISTR) tool. The SISTR platform is freely available online at https://lfz.corefacility.ca/sistr-app/ (accessed on 25 March 2021) [45]. The online tool (https://pubmlst.org/rmlst/, accessed on 27 September 2020) was used for the identification of ribosomal multilocus sequence typing (rMLST-types).

Additionally, with the aim of identifying the resistome, the virulome, and the Salmonella pathogenicity islands (SPIs), the draft genome was analyzed using ABRicate v1.0.1 (available online: https://github.com/tseemann/abricate/, accessed on 15 February 2021), software that includes different pre-downloaded databases, including ARG-ANNOT (doi:10.1128/AAC.01310-13) [46], NCBI (The NCBI Pathogen Detection Project. Bethesda (MD): National Library of Medicine (US), National Center for Biotechnology Information. 2016 May. Available online: https://www.ncbi.nlm.nih.gov/pathogens/, accessed on 20 March 2021; AMRFinderPlus (https://doi.org/10.1128/AAC.00483-19) [47], CARD (doi:10.1093/nar/gkw1004) [48], and ResFinder (doi:10.1093/jac/dks261). [49]. The presence of plasmid replicons was investigated using PlasmidFinder 3.0, available from the Center for Genomic Epidemiology (available online: http://www.genomicepidemiology.org/, accessed on 25 March 2021). Functional categories of the putative proteins encoded by the assembly were performed using the RPSBLAST 2.2.15 program on the orthologous groups of proteins COG database [50]. Clusters of orthologous groups (COGs) categories were recovered using the WebMGA software platform, with an e-value cut-off of 0.001 for prediction [51].

## 6. Conclusions

This study demonstrated the potential risks of *Salmonella enterica* causing extraintestinal infections in animals, highlighting the importance of continuous monitoring and surveillance, focusing on rare serotypes such as *S. houtenae*. WGS combined with epidemiological data may provide important information about the monitoring and surveillance of new, poorly described Salmonella strains. Massively parallel sequencing of these strains may help to identify clusters, AMR resistance determinants, virulence factors, and improve animal welfare.

## Figures and Tables

**Table 1:** Clusters of orthologous groups (COG) functional categories: class and number of genes identified in a *S. houtenae* isolate.

Class	Number of Genes	Function
J *	245	Translation, ribosomal structure, and biogenesis
A	25	RNA processing and modification
K	231	Transcription
L	238	Replication, recombination, and repair
B	19	Chromatin structure and dynamics
D	72	Cell cycle control, cell division, and chromosome partitioning
Y	2	Nuclear structure
V	46	Defense mechanisms
T	152	Signal transduction mechanisms
M	188	Cell wall/membrane/envelope biogenesis
N	96	Cell motility
Z	12	Cytoskeleton
W	1	Extracellular structures
U	158	Intracellular trafficking, secretion, and vesicular transport
O	203	Posttranslational modification, protein turnover, and chaperones
C	258	Energy production and conversion
G	230	Carbohydrate transport and metabolism
E	270	Amino acid transport and metabolism
F	95	Nucleotide transport and metabolism
H	179	Coenzyme transport and metabolism
I	94	Lipid transport and metabolism
P	212	Inorganic ion transport and metabolism
Q	88	Secondary metabolites biosynthesis, transport, and catabolism
R	702	General function prediction only
S	1347	Unknown

* Protein classification of the isolate based on the concept of orthology and one-letter abbreviations for the functional categories are used, as reported by the platform WebMGA.

**Table 2:** Antimicrobial-resistant (AMR) genes detected in the *S. houtenae* isolate.

Aminoglycoside	Multidrug		Fluoroquinolone	Nitroimidazole	Peptide	Fosfomycin	Aminocoumarin	ß-Lactam	Tetracycline
AAC(6′)-Iy	ampH	LptD	emrA	msbA	arnA	mdtG	mdtA	(Bla)AMPH E. coli	tet(34)
acrD	acrA	marA	emrB		ArnT		mdtB	(Bla)PBP *E. coli*	
kdpE	acrB	mdfA	emrR		bacA		mdtC		
	acrE	mdtM	mdtH		eptA				
	acrF	mdtN	mdtK		eptB				
	acrS	mdtO			yojI				
	baeS	mdtP			pmrF				
	baeR	OmpA			rosA				
	cpxA	OmpK37			rosB				
	CRP	ramA			ugd				
	golS	rsmA							
	KpnE	sdiA							
	KpnF	tolC							
	H-NS								

**Table 3:** Virulence factors detected in the *S. houtenae* isolate.

Virulence Factors												
Adherence		Magnesium Uptake	Secretion System			Stress Protein	Toxin	Pathogenicity Islands				
Agf	Type 1 fimbriae	MgtBC	TTSS (SPI-1 encode)	TTSS (SPI-2 encode)	Type VI effector	sodCI	spvB	SPI-1		SPI-2		SPI-5
csgA	fimC	mgtB	invA	pipB2	AHA_1833			invA	sicA	spiC/ssaB	ssaR	pipB2
csgB	fimD	mgtC	invE	sifA	fha			invB	sicP	ssaC	ssaS	sopB
csgC	fimF		invF	sifB	galF			invC	sipA/sspA	ssaD	ssaT	
csgD	fimH		invG	sopD2	hcp2/tssD2			invE	sipB/sspB	ssaE	ssaU	
csgE	fimI		invH	spiC/ssaB	tssA			invF	sipC/sspC	ssaG	ssaV	
csgG			invI	sseA	tssB			invG	sipD	ssaH	sscA	
			invJ	ssaD	tssJ			invH	spaO	ssaI	sscB	
			orgA/sctK	sscA	tssL			invI	spaP	ssaJ	sseA	
			prgH	ssaC	tssM			invJ	spaQ	ssaK	sseB	
			prgI	sscB	rhs/PAAR			orgA	spaR	ssaL	sseC	
			prgJ	sseB				orgB	spaS	ssaM	sseD	
			prgK	sseC				orgC		ssaN	sseE	
			sicA	sseD						ssaO	sseF	
			sipB/sspB	sseE						ssaP	sseG	
			spaO/sctQ	sseF						ssaQ		
			spaP	sseG								
			spaQ	sspH1								
			spaR	sspH2								
			spaS									

## Data Availability

The data presented in this study are available on request from the corresponding author.
